# Genetic structure in cultivated grapevines is linked to geography and human selection

**DOI:** 10.1186/1471-2229-13-25

**Published:** 2013-02-08

**Authors:** Roberto Bacilieri, Thierry Lacombe, Loïc Le Cunff, Manuel Di Vecchi-Staraz, Valérie Laucou, Blaise Genna, Jean-Pierre Péros, Patrice This, Jean-Michel Boursiquot

**Affiliations:** 1 Institut National de la Recherche Agronomique, UMR 1334 AGAP Amélioration génétique et adaptation de plantes, équipe DAVEM, 2 Place P. Viala, 34060, Montpellier, France; 2 INRA - Domaine de Vassal. Ancienne route de Sète, 34340, Marseillan Plage, France; 3 Institut Français de la Vigne et du Vin - Unité Mixte Technologique Géno-Vigne, 2 Place P. Viala, 34060, Montpellier, France; 4 Montpellier SupAgro, UMR 1334 AGAP Amélioration génétique et adaptation de plantes, équipe DAVEM, 2 Place P. Viala, 34060, Montpellier, France

## Abstract

**Background:**

Grapevine (*Vitis vinifera* subsp. *vinifera*) is one of the most important and ancient horticultural plants in the world. Domesticated about 8–10,000 years ago in the Eurasian region, grapevine evolved from its wild relative (*V*. *vinifera* subsp. *sylvestris*) into very diverse and heterozygous cultivated forms. In this work we study grapevine genetic structure in a large sample of cultivated varieties, to interpret the wide diversity at morphological and molecular levels and link it to cultivars utilization, putative geographic origin and historical events.

**Results:**

We analyzed the genetic structure of cultivated grapevine using a dataset of 2,096 multi-locus genotypes defined by 20 microsatellite markers. We used the Bayesian approach implemented in the STRUCTURE program and a hierarchical clustering procedure based on Ward’s method to assign individuals to sub-groups. The analysis revealed three main genetic groups defined by human use and geographic origin: a) wine cultivars from western regions, b) wine cultivars from the Balkans and East Europe, and c) a group mainly composed of table grape cultivars from Eastern Mediterranean, Caucasus, Middle and Far East countries. A second structure level revealed two additional groups, a geographic group from the Iberian Peninsula and Maghreb, and a group comprising table grapes of recent origins from Italy and Central Europe. A large number of admixed genotypes were also identified. Structure clusters regrouped together a large proportion of family-related genotypes. In addition, Ward’s method revealed a third level of structure, corresponding either to limited geographic areas, to particular grape use or to family groups created through artificial selection and breeding.

**Conclusions:**

This study provides evidence that the cultivated compartment of *Vitis vinifera* L. is genetically structured. Genetic relatedness of cultivars has been shaped mostly by human uses, in combination with a geographical effect. The finding of a large portion of admixed genotypes may be the trace of both large human-mediated exchanges between grape-growing regions throughout history and recent breeding.

## Background

Cultivated grapevine, *Vitis vinifera* subsp. *vinifera*, is one of the major horticultural crops worldwide. Domesticated from the dioecious taxon *V*. *vinifera* subsp. *sylvestris*[1-4], the modern grape is today more diverse and heterozygous than its wild relative [5-8].

The combined action of selection, breeding, admixture and migration is believed to have shaped the cultivated compartment, possibly starting from multiple gene pools during domestication [9,10]. Humans certainly selected traits related to fertility, blossom drop (*coulure*), productivity, berry size, sugar and acidity content [4,11,12], since these are keys for successful grape production. Similarly, hermaphroditism has been strongly selected for, almost to complete fixation, as self-pollinating plants achieve higher fruit production. Other traits were also probably selected, such as shoot habit, tolerance to biotic and abiotic stress, adaptation to local environment, and cuttings ability. Vegetative propagation is indeed believed to have been adopted early in the domestication process [11]. Large-scale grafting, in contrast, was only implemented at the end of the 19^th^ century, after the introduction of *Phylloxera* in Europe [13]. A slow selection process of promising local landraces early in domestication, followed by direct breeding and selection from the end of the Middle Age onwards, may have favored the emergence of family clusters of cultivars [14,15]. Finally, human-mediated movements of seeds and cuttings occurred even over long distances and were the means of grape extension to the New World, followed by selection and adaptation to local conditions [4]. The combination of such factors has most probably structured modern grapevine genetic diversity.

Up to now, studies searching for genetic structure in grapevine have been based on samples either relatively small (up to a few hundred) [5,9,10,16] or geographically limited [17]. Myles *et al*. [18], exploring SNP polymorphism in a set of 583 cultivars (404 of which had known geographic origin), found a weak East–west structure gradient; however their sample was under-representing some important regions (Caucasus, Spain, North Africa) and therefore could not be fully conclusive about genetic structure of the entire cultivated grapevine gene pool. Thus, the analysis of genetic structure in the largest grapevine collection available worldwide (INRA Vassal, France) could enable us to better understand the extent and distribution of grape diversity and how mankind shaped it.

In addition, structure analysis is a prerequisite for deciphering complex traits in genetic resources using association genetics, a methodology that already yielded interesting results in grape [19,20]. The extensive diversity of grapevine [8] and recent progresses in DNA analysis technologies make genome-wide association genetics over hundreds of accessions the next target for grapevine research. Therefore, population structure analysis is today all the more pivotal.

The genetic material maintained at the French grapevine collection of Vassal [21] has been built up over the last 140 years through a large network of international partnerships. More than 5,000 accessions of *Vitis vinifera* have been morphologically and agronomically characterized [22] and historically documented. Using twenty microsatellite markers at linkage equilibrium [23], Laucou *et al*. [8] identified 2,323 unique genotypes in this collection. Microsatellite markers (or Simple Sequence Repeats, SSR) are indeed suitable to better understand the genetic structure of cultivated plants [24-26].

The main objectives of the present study were to understand the genetic diversity of cultivated grapevine and how humans molded it over the years, and to provide a description of genetic structure that could be used to select genotype samples appropriate for further genetic association studies. We used a subset of 2,096 microsatellite genotypes from the Vassal database, without missing data and with at least two allele differences. Statistical tests were conducted to control the possible effects of sampling, in particular the bias that could arise from oversampling one geographical region or one family group.

We analyzed the structure using two different methods of clustering, the first based on a Bayesian approach [27], and the second using Ward’s hierarchical clustering method [28,29]. We also compared the genetic diversity and family relatedness within the clusters at different levels of ancestry. Finally, to interpret population structure in relation to both cultivar use and history, we characterized the clusters using the geographic origin of cultivars and their phenotypic characteristics.

## Results

### Diversity

Our sample of 2,096 cultivated genotypes (Additional file 1: Table S1) displayed from 5 to 34 alleles per locus, with a total of 324 alleles over the 20 loci, an unbiased expected heterozygosity (He) of 77% and a mean polymorphism information content (PIC) of 0.740 (Additional file 2: Table S2). Owing to the large number of loci with moderate allele frequencies, the single parent exclusion probability was quite high (7 × 10^-6^).

### Population structure

**Figure 1:**
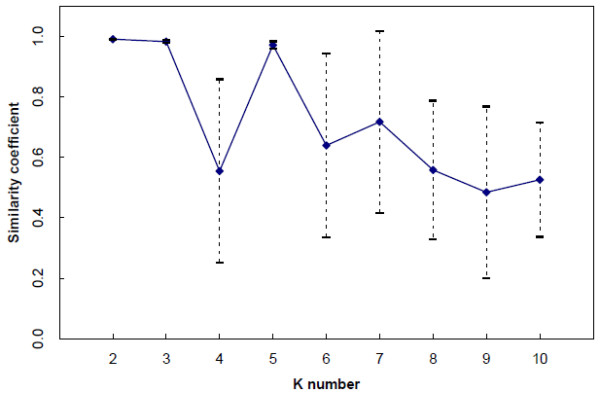
**Similarity index among STRUCTURE runs.** Similarity index among runs for each STRUCTURE K-level, and its confidence intervals (10 runs for each K).

The similarity pattern among the 10 STRUCTURE analysis replicates (Figure 1) and Evanno’s ΔK_s_ statistics (Additional file 3: Figure S1) indicated K_s_ = 3 and K_s_ = 5 as the most pertinent levels of population subdivision. No converging solutions were identified for the subsequent Ks levels (6 to 12), which were therefore not further considered with STRUCTURE.

Using a threshold of >85% for group assignation, 1,001 genotypes (out of the 2,096) were assigned to a cluster at K_s_ = 3 and 817 at K_s_ = 5. The proportion of admixed genotypes was thus large, *i*.*e*. 52% and 61% of the total number of cultivars, at K_s_ = 3 and K_s_ = 5 respectively.

Since the repartition of genotypes from the different regional groups was not equilibrated (Table 1), we tested STRUCTURE sensitivity to the effect of sampling, comparing the full genotype collection (*set 1*: 2,096 genotypes) to a subset of *set 1* with equilibrated geographic groups (*set 2*: 888 randomly chosen genotypes, Table 1). Genotype partitioning in STRUCTURE subgroups was stable between the two different samplings, the individual K-scores for *set 2* being almost perfectly correlated to those of *set 1* for K_s_ = 3 (r^2^ = 0.97, p < 0.0001) and K_s_ = 5 (r^2^ = 0.98, p < 0.0001). Instabilities among repeated runs were found at K_s_ = 4 and K_s_ = 6, both within and between *set 1* and *set 2*. Because of these coherent results, all subsequent analyses were based on the full set of 2,096 genotypes (*set 1*).

**Table 1:** Composition of the cultivar groups used in the analysis

**Group Name**	**Group Code**	**Subgroup Name**	**Subgroup Code**	**Countries**^**a**^	**Number of cultivars**	**Number of cultivars for testing sampling effect**^**b**^
Maghreb	MAGH	Maghreb	MAGH	DZA, MAR, TUN	83	83
Iberian Peninsula	IBER	Iberian Peninsula	IBER	ESP, PRT	226	100
Western & Central Europe	WCEUR	Western Europe	WEUR	BEL, FRA, GBR, NLD	567	100
		Central Europe	CEUR	AUT, DEU, CHE, CZE, SVK, TCH	111	100
Italian Peninsula	ITAP	Italian Peninsula	ITAP	ITA	304	100
Balkans	BALK	Eastern Europe	EEUR	BGR, HUN, ROU	205	100
		Balkan Peninsula	BALP	BIH, CYP, GRC, HRV, SCG, YUG	149	100
Russia & Ukrain	RUUK	Russia & Ukrain	RUUK	MDA, RUS, UKR, URS	94	94
Eastern Mediteranean & Caucasus	EMCA	Caucasus & Turkey	CAUC	ARM, AZE, GEO, TUR	89	89
		Near East	NEAS	EGY, ISR, LBN, SYR,	51	51
Middle & Far East	MFEAS	Middle East	MEAS	IRN, YEM	29	29
		Central Asia & Far East	FEAS	AFG, CHN, IND, JPN, KAZ, TJK, TKM, UZB	53	53
New World Vineyard	NEWO	New World Vineyard	NEWO	ARG, AUS, CHL, MEX, PER, USA, ZAF	106	100
Non determined	ND	Non determined	ND		29	29

^a^ According to the ISO 3166–1 alpha 3 codes (http://en.wikipedia.org/wiki/ISO_3166-1_alpha-3). ^b^ Sub-sample build with the aim to study a possible effect of differences in sample size among geographic regions, on *STRUCTURE* analysis.

Countries of origin were grouped into larger regional groups, for easiness of representation and statistical tests.

### Geographic origin and viticultural traits of the identified subgroups

**Figure 2:**
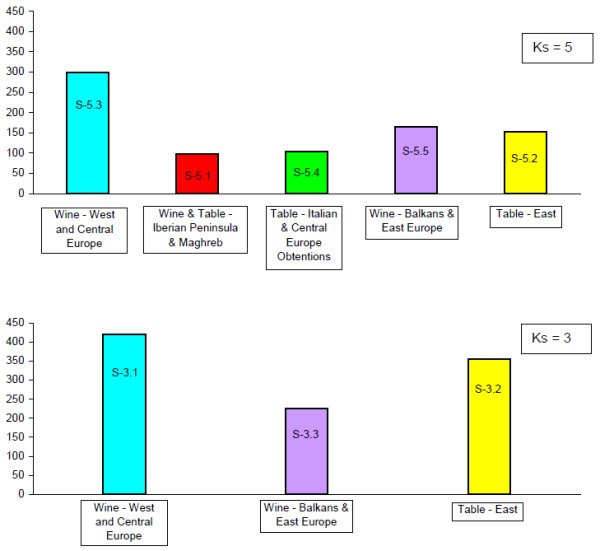
**Characterization of the STRUCTURE groups.** Characterization of the STRUCTURE groups according to geography and use.

STRUCTURE clustering at K_s_ = 3 highlighted three well-distinct groups (Figure 2, Additional file 4: Table S3):

a Western Europe group (S-3.1) of wine cultivars (93%) containing 55% of the Western and Central Europe genotypes, without any Asian, Balkans or Maghreb cultivars;

a East group (S-3.2) mostly composed of table cultivars (71% of table grapes and 9% of ‘double-use’ cultivars), including 96% of the Far- and Middle-East genotypes, notably all genotypes from Uzbekistan (n = 33), Afghanistan (8), Tajikistan (4), Turkmenistan (4) and Iran (23), as well as 66% of the Eastern Mediterranean and Caucasus cultivars, and almost no Western and Central Europe cultivars (less than 2%). Interestingly, 43% of the cultivars from Maghreb were positioned within this group;

a Balkan and Eastern Europe group (S-3.3) of mostly wine cultivars (71%), with 34% and 60% of the varieties from the Balkans and East Europe, and less than 4% Western Europe cultivars.

**Figure 3:**
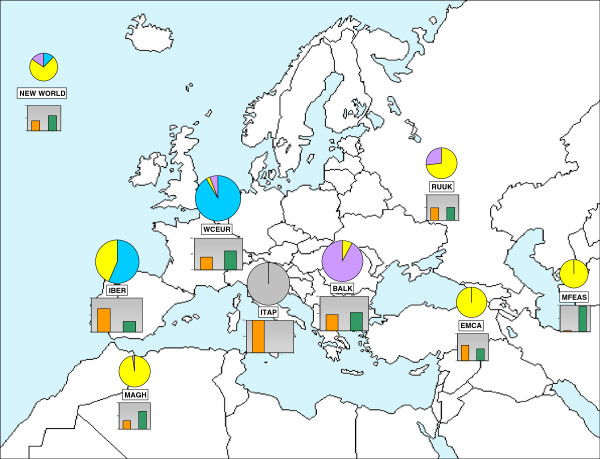
**Genetic composition of the geographic groups.** Genetic composition of the geographic groups for the K*s* = 3 of STRUCTURE. For the detailed country list, see Table 1. The histograms represent the percentage of non-admixed (green) versus admixed (orange) genotypes. For the non-admixed cultivars, the pies represent the proportion of each cluster in each region: Table / East (yellow); Wine / Balkans and East-Europe (violet); Wine / West and Central Europe (blue). As 100% of the Italian genotypes are admixed, the ITAP pie is empty (grey).

The relationship between the different clusters and cultivar geographical origin was analyzed (Figure 3). The Eastern Mediterranean & Caucasus (EMCA), Middle & Far East (MFEAS) and Maghreb (MAGH) geographic groups (Table 1) were composed almost exclusively of genotypes clustered in the S-3.2 STRUCTURE genetic group; the Western & Central Europe (WCEUR) and the Balkans (BALK) geographic groups were also mainly composed of genotypes from their corresponding STRUCTURE group (S-3.1 and S-3.3, respectively). By contrast, the Russian, Iberian and New World cultivars were distributed in several STRUCTURE genetic groups. For K_s_ = 3, it is worth noting that 100% of the Italian cultivars were assigned to the “admixed” class, while the Middle and Far East group displayed a very low level of admixture (3.6%). Apart from its meaningful geographic distribution, the admixed group was composed of even proportions of wine or table cultivars, of black, red or white color grapes, and of aromatic or non-flavored grapes.

The clustering at K_s_ = 5 (Figure 2, Additional file 4: Table S3) identified in addition an Iberian and Maghreb group (S-5.1), and a group comprising mostly table grapes (80%) of recent origin, also called “obtentions”, from Italy and Central Europe (S-5.4). The group S-5.1 derived partially from the S-3.2 group (41% of the varieties), with Iberian varieties composing 69% of the group. The group S-5.4 mostly derived from the admixed Ks = 3 group (78% of the varieties).

**Figure 4:**
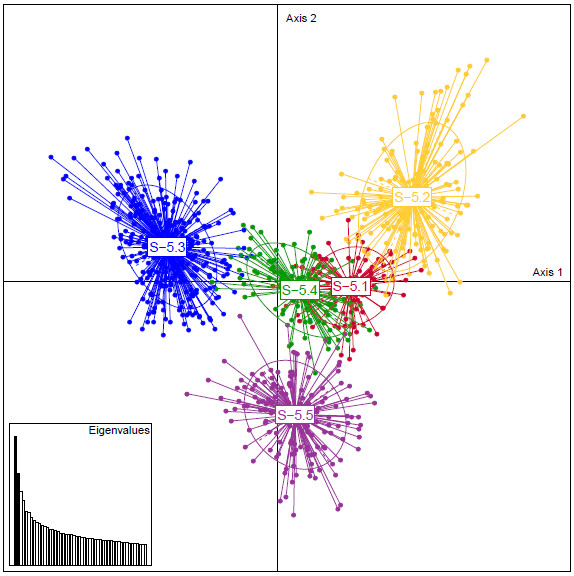
**Principal component analysis on SSR data.** Principal component analysis on SSR data of genotypes belonging to Ks = 5. Colors of the groups correspond to the colors in Figure 2. Axes 1 and 2 explain 30.3% and 21.4% of the total variance respectively (black vertical bars in the eigenvalues histogram).

Axes 1 and 2 of a PCA on SSR data of the genotypes belonging to Ks = 5, explaining 30.3% and 21.4% of the total variance respectively (Figure 4), clearly separated the WCEUR, BALK and East groups. The two additional groups at (S-5.4 and S-5.1) were separated by PCA only on axes 3 and 4 respectively (not shown), which explained 14.3% and 8.8% of the total variance. Thus genetic clustering at K_s_ = 3 appeared more structuring than the one at K_s_ = 5.

**Figure 5:**
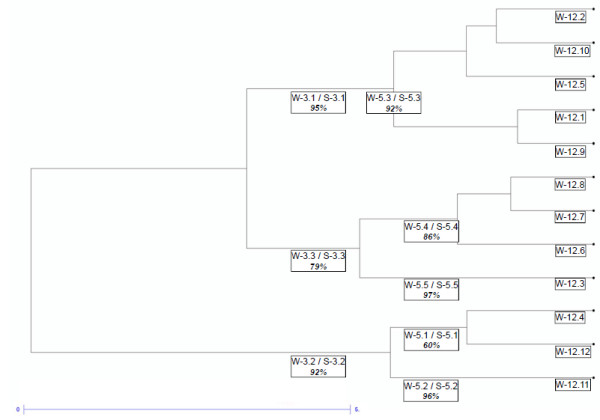
**Dendrogram based on Ward**’**s clustering.** Dendrogram based on Ward’s clustering. For levels three and five of clustering, the comparison among the Ward and STRUCTURE groups is summarized with the double code labels (W-x.x / S-x.x), and the percentage of shared individuals between them.

To support the STRUCTURE analysis, we performed a Ward clustering (Figure 5; the full dendrogram for the 2,096 cultivars is given in Additional file 5: Figure S2). The Ward and STRUCTURE clustering were found consistent (Additional file 6: Table S4), with a correspondence among clusters composition of 90% and 87% for K = 3 and K = 5 respectively. In addition to the main partitions already explored at Ks = 3 and 5, the Ward clustering level at K_w_ = 12 (Additional file 7: Table S5) identified local germplasms, groups of cultivars with a particular characteristic (white, seedless or muscat flavor grapes), or parentages linked to human selection and breeding (next paragraph).

### Genetic diversity and family structure within and among clusters

The genetic diversity of the K_s_ = 3 and K_s_ = 5 groups is described in Table 2. In both cases the Table - East group, although not the largest one, was by far the most diverse, displaying the largest number of common and private alleles, as well as the largest non-biased heterozygosity. Observed and expected non-biased heterozygosity statistics were generally large but not significantly different among groups. No significant heterozygosity deficit could be detected (not shown).

**Table 2:** **Statistics of genetic diversity for the STRUCTURE sub**-**populations at Ks** = **3 and 5**

**K**_**s**_**level**	**K**_**s**_**group**	**N**	**Mean number of alleles** / **locus**	**Private alleles** (**absolute count**)^**a**^	**Private alleles** (**equal samples**)^**a**^	**Ho**	**He**^**b**^	**Standard deviation He**
**Ks** = **3**	S-3.1 (Wine - West & Central Europe)	419	9.95	9	1.07	0.751	0.715	0.154
	S-3.2 (Table - East)	356	12.65	19	2.96	0.742	0.753	0.126
	S-3.3 (Wine - Balkans& East Europe)	226	8.9	0	0.28	0.734	0.71	0.132
**Ks** = **5**	S-5.1 (Wine & Table - Iberian Peninsula & Maghreb)	97	7.6	1	0.38	0.73	0.69	0.159
	S-5.2 (Table - East)	153	10.95	22	2.23	0.75	0.76	0.097
	S-5.3 (Wine – West and Central Europe)	298	9.15	4	0.64	0.74	0.7	0.139
	S-5.4 (Italy & Central Europe)	104	6.35	1	0.07	0.73	0.68	0.143
	S-5.5 (Wine - Balkans & East Europe)	165	8.3	1	0.29	0.73	0.7	0.134

^a^ Private alleles are displayed both as absolute count values in the whole sub-populations (if allele frequency > 0.01), and adjusted for sample bias, considering equal-sized sub-samples, as in Zachary *et al*. [58].

^b^ He represents the non-biased heterozygosity as in Nei [71].

The average genetic differentiation among STRUCTURE groups for K_s_ = 3 and K_s_ = 5 was D_*est*_ = 0.166 and 0.213 respectively (harmonic means; in both cases, standard deviation D_*est*_ = 0.005). The largest differentiation between pairs of subpopulations was found between the Western-Central Europe and the Eastern groups, for both K_s_ = 3 and 5, with D_*est*_ = 0.217 and 0.256 respectively). The Eastern group and the group from the Iberian Peninsula and Maghreb displayed a D_*est*_ of 0.139, the lowest of all comparisons (Additional file 8: Table S6).

Since the presence of family groups may affect population genetic structure, we explored the distribution of cultivar family relationships within and among STRUCTURE groups. By comparing all possible genotype pairs, the ML-relatedness software outlined in total 1,069 likely parent pairs involving 1,099 putative parents.

The distribution of the likely parent pairs among and within STRUCTURE (K_s_ = 3 and 5) and Ward (K_w_ = 12) groups is shown in Table 3. The two members of a family-related pair were more frequently found within the same cluster than in different clusters; the coefficient of relatedness was also significantly higher within clusters compared to whole population relatedness (*r*^*2*^ = 0.046). Smaller relatedness values were found within the admixed group or at the margins of the STRUCTURE clusters (one parent belonging to a cluster and the other to the admixed group). In the admixed group, we found 3.0% and 3.2% of genotypes with parents in two different STRUCTURE groups for K_s_ = 3 and K_s_ = 5, respectively (not shown). The parentage at the inter-K level was not significantly different from the average parentage in the entire population.

**Table 3:** **Distribution of family relationship among genotype pairs**, **classified within and between structure subgroups**

**Structure level**	**Level**	**Related pairs** (**HS**, **FS**, **PO**)	***r***^**2**^
K_s_ = 3	Intra-Clusters	47%	0.11
	Inter-Clusters	1%	0.02
	Intra-Admixed	24%	0.05
	Inter Cluster/Admixed	28%	0.04
K_s_ = 5	Intra-Clusters	39%	0.14
	Inter-Clusters	1%	0.03
	Intra-Admixed	27%	0.04
	Inter Cluster/Admixed	33%	0.04
K_w_ = 12	Intra-Clusters	63%	0.14
	Inter-Clusters	37%	0.04

Note: For each couple, if both genotypes belonged to the same K group, or to the admixed group, the couple was assigned to the Intra-Clusters or the Intra-Admixed classes, respectively; similarly, a couple was assigned to the Inter-clusters and Inter Cluster/Admixed classes if they belong to different Ks or if one belong to one K and the other is “admixed”, respectively.

Ward’s clustering does not identify the “admixed” class.

At the Kw = 12 subdivision level, the analysis of the family structure (percent of genotypes with family relations within group and average within-group relatedness) allowed to characterize and confirm four already described kin groups, essentially formed by one family (Table 4), and identified two additional groups composed by a mix of several families. One example was the W-12.6 group, with 48% of all genotypes related to each other, a relevant proportion of which were recently bred white table grapes with muscat flavor. On the opposite, the W-12.12 group, comprising Caucasian, Russian and Maghreb cultivars (derived from the S.3.2 East table grapes group) displayed the lowest relatedness and the smallest number of family-related genotypes.

**Table 4:** **Characterization of the clusters at K**_**w**_ = **12**, **based on their genetic diversity**, **family relationship and phenotypic characteristics**

**Group Code**	**N**	**He**	**Percent of genotypes with family relations in the same group**	**Average relatedness** "**r**" (**st dev**)	**Description of the main family composing the group**	**General group description**
W-12.1	99	0.65	51%	0.26 (0.039)	Family group of Gouais blanc	Black and white wine cultivars from Western Europe
W-12.2	157	0.71	43%	0.16 (0.027)	Family groups of Savagnin and Cabernet franc	Black and white wine cultivars from South West of France and Portugal
W-12.3	220	0.71	35%	0.12 (0.029)		Wine cultivars from Central Europe and Balkan peninsula, with mostly white, red, grey or pink berry skin color
W-12.4	169	0.72	30%	0.11 (0.015)		Black and white wine cultivars from Iberian Peninsula and Maghreb.
W-12.5	136	0.76	12%	0.04 (0.013)		Black and white cultivars (mostly for wine but also for table grape) from Italy and Alps.
W-12.6	128	0.71	48%	0.13 (0.020)	Mix of several recently bred families	Table grape cultivars, new breeding or modern selections, with mostly white berry skin color and muscat flavor.
W-12.7	127	0.70	27%	0.14 (0.017)	Several families obtained by breeding in the XIXth and early XXth century	Traditional breeding of table grape (including some cultivars with muscat flavor) selected in several countries during XIXth century and early XXth.
W-12.8	105	0.71	70%	0.15 (0.018)	Family groups of Chasselas and Muscat à petits grains	Wine cultivars with white, red, grey or pink berry skin color. Presence of several cultivars with muscat flavor
W-12.9	107	0.70	50%	0.16 (0.024)	Family group of Pinot and Riesling	A majority of white wine cultivars from Western Europe
W-12.10	220	0.74	21%	0.06 (0.014)		Wine cultivars with mostly black berry skin color, from Italy (Centre and North), France (Alps and South), Spain (North) and Portugal.
W-12.11	276	0.75	38%	0.07 (0.018)		Traditional table grape cultivars originated from Far, Middle and Near East, with mainly white, red, grey or pink berry skin color ; this group gathers most of the seedless grapevine cultivars
W-12.12	352	0.76	14%	0.03 (0.014)		Diverse wine and table grape cultivars in particular from Caucasus, Russia and Maghreb

N is the number of genotypes within the group; He represents the non-biased heterozygosity as in Nei [71]; the average relatedness “*r*” is calculated with the Queller and Goodnight formula [72], and the standard error using 100 jackknifes over loci [30].

All the analyses above allowed us to characterize the different groups (Table 4 and Additional file 9: Table S7) for their genetic diversity, family relationships and phenotypic characteristics, and finally propose an interpretation of grapevine genetic history and geographic partitioning.

## Discussion

The main objective of this study was to explore the genetic diversity and structure of cultivated grapevine and link them to cultivar utilization, putative geographic origin and historical events. Microsatellite markers’ data for 2,323 unique cultivars collected and maintained at the French grapevine collection of Vassal (INRA, France), were available [8]. Inferences of population structure were derived with both a Bayesian and a hierarchical clustering method. Since clustering methods may be sensitive to sampling bias, to improve our chances to detect true structure patterns, we followed three strategies, i) first we focused only on the 2,096 genotypes (out of the 2,323 unique cultivars) without missing SSR data and excluding putative clones and close mutants (with only one or two allele differences over the 40 alleles); indeed, missing data may bias the clustering procedure, and nearly identical SSR genotypes can be considered as redundant for our scope; ii) secondly, we evaluated the possible bias due to unbalanced geographical representativeness of our sample, by running STRUCTURE analysis on two data sets, one with the entire sample and the other balanced in term of cultivar geographical origin (cultivars being randomly picked within each geographical group). STRUCTURE provided a very consistent attribution of genotypes to clusters independently of the data set, thus only the full set of genotypes was further analyzed; iii) third, since the STRUCTURE clustering method can be disputed because human manipulation of cultivars (displacements, breeding, clonal propagation) could have generated a deviation from the Hardy-Weinberg equilibrium, we complemented the STRUCTURE analysis with the method of discriminant analysis developed by Ward, which is independent from any assumptions on population dynamics. According to Odong *et al*. [30], the two methods are complementary, so they can conveniently be used together and compared.

The 2,096 cultivars of the Vassal collection studied here originate from 52 countries around the world, making our sample highly representative of the cultivated grapevine gene pool. Our data confirmed the high levels of diversity and heterozygosity of the cultivated grapevine, in agreement with a number of previous studies [4,5,8,17,18]. This can be due to an intermix of factors: i) a weak bottleneck effect during domestication [18] as observed for maize and wheat [31,32], probably in relation to ii) vegetative propagation and diffusion of cuttings across geographic regions [11], iii) several putative domestication events from different gene pools [9,10], then intermixed by man with breeding and selection, and iv) diversifying selection in plant breeding [33]. The large diversity found in grapevine opens an avenue for further selection and breeding [18]. Among the 2,096 genotypes studied here, over one half is still poorly known from a viticultural and oenological point of view and may potentially carry new genes and traits of interest for new breeding and selection.

STRUCTURE identified one main level of population subdivision at K_s_ = 3 and a secondary subdivision at K_s_ = 5. A PCA analysis and Ward’s hierarchical clustering confirmed this finding. Both the STRUCTURE and Ward methods indicated inconsistencies in clustering for K = 4 and 6, suggesting that these two levels are not appropriate for subdividing the grapevine gene pool. While confirming the main subdivision, Ward’s clustering also pointed to a finer structure linked to grapevine uses, family structure or local geographic groups.

The analysis of family relationships also revealed that STRUCTURE clustered a significant portion of family-related genotypes, nearly double of the fraction found in the admixed group. By contrast almost no parentage was found among genotypes from different K3 groups (inter-group level). These findings are probably the result of the history of grapevine, with the practice of breeding focusing mostly on local varieties.

In the admixed group we could identify approximately 3% of genotypes with parents classified in two different STRUCTURE clusters, such as the wine grape Tarrango, known to be a cross between Touriga (a wine grape from Portugal, S-3.1 group) and Sultanina (a seedless table grape from Turkey, S-3.2 group). The crossing among genotypes from different STRUCTURE groups probably corresponds to recent breeding activity in search for novelties and hybrid vigor, remaining nevertheless proportionally marginal.

We also detected significantly more family relationships within the already know grapevine kin groups of i) Gouais [15,34,35], ii) Savagnin and Cabernet franc [14], iii) Chasselas and Muscat, and iv) Pinot and Riesling [36], and found traces of existence of two additional groups, each composed by a mix of several families, such as the W-12.6 and W-12.7 groups, comprising family-related table grapes with muscat flavor released by modern breeding.

The interaction of genetic structure and family relationship is known to be difficult to resolve, and 20 microsatellite loci are probably not sufficient to avoid false positives, despite the large number of alleles. Nevertheless, our family relationship analysis, seen as a tentative to understand large scale population patterns and not to precisely detect each single family pair, provided a coherent global picture. This analysis was also coherent with a more specific paper by Lacombe *et al*. in 2012 [37] who explored direct parentage using an exclusion probabilities algorithm, with a slightly different sample, thus explaining minor differences.

### Geography and history

The three main clusters revealed by our study, both with STRUCTURE and Ward’s methods, confirmed previously obtained molecular results [5,9] and the eco-geographic grouping proposed by Negrul [38], in particular the correspondences between the “*proles*” *occidentalis* and S-3.1/W-3.1 groups, the *pontica* and S-3.3/W-3.3 groups, and the *orientalis* and S-3.2/W-3.2 groups. Our results allow us to subdivide these clusters according to cultivar putative geographical origins: i) West and Central Europe (S-3.1), ii) East Mediterranean, Caucasus, Middle and Far East (S-3.2), and iii) Balkans and East Europe (S-3.3). Clustering at K = 5 identified two new groups, an Iberian Peninsula group and a group of table grape obtentions with Italian Peninsula and Central Europe origins.

Genetic characterization of the groups clearly showed the East table grape group (S-3.2 and S-5.2 for K = 3 and 5 respectively) as the most diverse in terms of mean number of alleles, number of private alleles, and non-biased heterozygosity. This is consistent with the hypothesis that grapevine domestication initially occurred in Eastern regions (Caucasus and Fertile Crescent) as suggested earlier [2-4,9], repeatedly introducing genes from the wild. The high frequency of private alleles in S-3.2 and S-5.2 could also be explained by a history of limited exchanges from East to West, as attested by the high differentiation values (D_est_) between these regions, and a slower development of grape breeding in the East, as indicated by the low frequency of family-related genotypes in that region as compared to other regions, revealing a weaker selection bottleneck effect there. However, given the high genetic diversity of grapevine at all subdivision levels, the selection and breeding bottlenecks seem in general weak for this crop.

The second most diverse group was the West and Central Europe wine grape group, probably as a result of this area’s long history of grapevine cultivation and development, in combination, as already stated by other authors, with gene flow from local wild or primo-domesticated grapevines [9,10,18]. The Balkans and East Europe cluster also formed a well identified STRUCTURE group with an intermediate diversity. The two additional groups at Ks = 5 (the Iberian Peninsula group and the group of table grape obtentions), appeared as secondary groups with a lesser global diversity.

More generally, the full hierarchical partitioning obtained with the STRUCTURE and Ward methods as well as the D_est_ differentiation statistics appeared consistent with historical data, such as the diffusion of viticulture around the Mediterranean Sea, with one route connecting Eastern (W-3.2) to Western Europe through the Balkans and Central Europe (W-3.3, W.3.1) [2,9], and a Southern route to the Maghreb and Iberian peninsula (W-3.2 /W-5.1 / W-12-4).

The Balkans and Eastern Europe group and the Western and Central Europe group were both characterized by a large proportion of genotypes belonging to one STRUCTURE group only, probably corresponding to separate regional grapevine cultivar development and selection. In contrast, other regions as Russia and Ukraine, the Iberian Peninsula, and the New World countries, contain a mix of two or three STRUCTURE groups, in relation to their regional position. In particular, varieties found in Russia and Ukraine appear to have either East (S-3.2), Balkans and East Europe (S-3.3) origins, consistently with what we know of the centralizing impact that Russian agricultural research had during the Soviet period [39]. Similarly, the Iberian peninsula group include cultivars from West Europe (S-3.1), East (S-3.2) and Maghreb (S-5.1) as well as a high proportion of admixed genotypes, in coherence with the long historical exchange relationships this region had both with Europe and North Africa. Based on maternally inherited chloroplast markers, Arroyo-Garcia *et al*. suggested that the Iberian Peninsula could be a secondary center of domestication [9]. Our results add a new view of Spain and Portugal as platforms of centralization, intermixing and exchange of varieties throughout history.

Finally, at K_w_ = 12, the genotypes from the eastern regions (*proles orientalis*[38]) further subdivided into two sub-groups, one mainly composed of wine cultivars of Caucasian origin (including Georgia, Armenia, Azerbaijan and Turkey, W-12.12), and the other comprising table cultivars from Central Asia (Tajikistan, Uzbekistan, Turkmenistan) together with Iran and Afghanistan (W-12.11). The separation of these two groups may be a trace of divergent selection for the main local use for grapevine (table *vs*. wine). On the other hand, the absence of admixture in the Middle and Far East group, in particular for the 72 cultivars from Uzbekistan, Afghanistan, Tajikistan, Turkmenistan and Iran, and the high K scores of its genotypes may be an indication that the corresponding center of domestication was larger than formerly believed (several authors indeed placed it in a geographic region between the Black Sea and Iran [2,3,40,41]), an hypothesis already proposed in 1976 by Olmo [12], but not confirmed by later studies. It is difficult to decide between these two scenarii since the information available on grapevine crop development is quite limited for Central Asian countries.

A large proportion of admixed genotypes was found by STRUCTURE, both at K_s_ = 3 and K_s_ = 5. A previous study on maize indicated that, in crops, STRUCTURE grouping is generally coherent for first cycle inbreds with simple parentage relationships, while the presence of multiple levels of family relationships and cohort overlapping in more advanced breeding systems leads to different grouping possibilities and low STRUCTURE stability [42]. We can infer that our sample contains both types of material, with a number of ancient varieties anchoring the main clusters (founders), and recent breeds complicating structure resolution. The stability of K_s_ = 3 and K_s_ = 5 groupings and the individual percentage of cluster ancestry allowed us to discriminate among these two types of materials. The geographic distribution of the admixed genotypes is not “random” (Table 1): the Middle-Far East is the region displaying the lowest level of admixture, while Italy in particular and secondly the Iberian Peninsula, display the larger proportion of admixed genotypes. We were unable to find other traits characterizing the admixed group: it is composed of even proportions of phenotypic classes of grape use, berry color, flavor, berry seed number, or sex.

While confirming and reinforcing the observation of geographic structure of the cultivated gene pool already described by other authors [5,9,16,18,38], our results are also coherent with the study of Cipriani *et al*. [17] suggesting that Italian varieties present weak or no structure: indeed in our study the Italian cultivars appear to be admixed, probably as a result of the inter-regional exchange role that Roman culture has certainly played.

### Phenotypic traits

Our results also provide information about the effect human selection on morphological traits had on shaping the genetic diversity of cultivated grapevine. Table and wine grapes have different berry size and bunch shapes, both important traits used for cultivar classification [22]. Table and wine grapes are clearly separated by STRUCTURE at *K*_*s*_ = 3. At *K*_*s*_ = 5; only the group including Iberian and Maghreb cultivars (S-5.1) is composed of a mix of table and wine cultivars, which is likely the result of artificial selection and intimate cultivars intermixing in this area.

The black color of berries is considered as an ancestral trait compared with the other colors, both at phenotypic [1] and molecular level. The molecular basis of the apparition of red, rose, grey and white berry colors has been previously documented [43-45] and the diffusion of the major causal mutations – *Gret*1 insertion and K980 mutation – within the cultivated compartment was described by Fournier-Level *et al*. [19]. In the present STRUCTURE analysis, the Central and West Europe subgroup (S-5.3) is composed of a majority of black cultivars. This can be explained by the isolation of these regions from the Eastern cultivars, by local domestication and gene flow from endemic black-berried *V*. *v*. *sylvestris*, or human selection. All other subgroups include a large number of white cultivars, reinforcing the idea of a wide and strong diffusion of *Gret*1 over the whole geographic range of grapevine [19]. Most of the intermediary phenotypes (red, rose and grey) are concentrated within two groups: Balkans and Central Europe (S-5.5), and East (S-5.2), confirming these regions as putative sources of color variation [19].

The geographical origin of Muscat flavor is assumed to be Greece or the Balkan Peninsula [46,47]. Thereafter, human selection aimed to spread this desirable trait in both table and wine grapes [20]. With STRUCTURE, we found the majority of Muscat founders within the Central Europe table group (S-5.4). Only a small number of them were involved in breeding, essentially in the Balkans, forming kingroups with other known parents such as Chasselas.

Seedless cultivars clustered essentially with cultivars of Turkish, Caucasian and Asian origins, belonging to the *proles orientalis*[38], coherently with available historical data about their origins from Turkey and Near-East [48].

## Conclusions

The array of analytical methods used here contributed coherent information to interpret the geographic and phenotypic structure, pointing to the main differentiation axes that exist within the cultivated compartment of *Vitis vinifera*, as it was shown in other species, such as potato [49], poplar [50], and maize [51]. Archaeological and historical data suggest that domestication of grapevine took place in the region spanning from the Fertile Crescent to South Caucasus [2,3,40,41], and from there spread in three directions: a Northern route, through Greece and the Roman empire to its western borders; a Southern route, through Egypt, the Arab territories all the way to Spain during the last Arab invasions; and a third route towards Asia. The results of our analysis, in particular the three groups defined by K_s_ = 3 and the finding of intermixed resources in focal regions of grapevine development, are consistent with this historical scenario. New elements provided by our studies are: i) the identification of the Iberian and Italian Peninsulae as regions of intermixing and exchange of varieties; ii) an East–west bottleneck effect due to limited cultivar migration, complemented by local selection and breeding; iii) a more extended center for grapevine primo-domestication, expanded to Central Asian countries.

Our results also suggest that signs of weak genetic structure in grapevine found by earlier studies were probably due to either regionally and/or numerically limited sampling. By examining the molecular data on the large INRA grape collection of Vassal, we have shown that grape diversity is structured into groups that interestingly reflect historical evolution, migration and human selection.

The subpopulations identified in this study, and in particular at K_*s*_ = 3 or K_*s*_ = 5, will be very useful to define samples for linkage disequilibrium and genetic association studies [52], especially for traits of significance for local adaptation or sensitive to local human selection. The finding of large proportions of family relationships within structure groups should however be taken in account. Genetic association studies may certainly be optimized by the use of the structure and parentage matrices as covariates in the analysis.

In the near future, the study of additional cultivars from regions less represented in our sample (such as the Caucasus area), the study of wild genotypes from around the distribution area including Central Asian countries, and the use of other markers associated with genes of interest linked to domestication and selection processes, will provide more precise information about the evolution of *Vitis vinifera*.

## Methods

### Plant material

The plant material was composed of cultivated grapevine varieties belonging to *V*. *vinifera* subsp. *vinifera* held in the INRA grape repository at Vassal (France). This collection includes 3,727 accessions available as field-grown plants and genotyped with 20 microsatellite markers [8].

### Geographic assignation

Geographic origin of referenced cultivars was derived from general bibliography on ampelography and viticulture [53,54]. For non-referenced cultivars, the origins were estimated on the basis of the accession origin. Recently bred cultivars (e.g., Tarrango) were assigned to the breeder’s country (in this example, Australia) and not to the countries of origin of their progenitors (in this case, Portugal and Turkey). Countries of origin were grouped as shown in Table 1.

### Cultivar characterization

Cultivar traits were observed and recorded over several years, using the methods developed for the grapevine genetic resources catalogues [53-55], and coded according to the International Organization of Vine and Wine descriptors [56], as presented in Table 5.

**Table 5:** **List of the phenotypic traits recorded for group characterization and coded according to the OIV** (**2009**) **notation system**

**Characteristic**	**Level of expression**	**International Code**
Utilization of the fruit	Wine grape (W); Table and/or raisin grape (T).	-
Berry skin color	White (B); Black (N); Rose (Rs); Grey (G); Red (Rg).	OIV-225
Berry flesh color	Non-colored (NoC); Colored (Col).	OIV-231
Berry flavor	None (No); Muscat (Mus); Herbaceous (Herb); Other (Oth).	OIV-236
Presence of seeds	Seeded berry (SD); Seedless berry (SL).	OIV-241
Sex of flower	Hermaphrodite (H); Female (F).	OIV-151

### DNA extraction and genotyping

DNA extraction, PCR amplification and genotyping of microsatellites were carried out according to This *et al*. [4] and Laucou *et al*. [8]. The 20 nuclear microsatellite loci were chosen according to their polymorphism level and their position on the linkage groups [23]. Differences of one base pair between alleles at one given locus were double-checked by re-amplification and re-analysis; a test for the presence of null alleles was also carried out [57].

Since genotypes with only one or two allele differences represent closely related material, such as clones or recent mutants, and provide very little additional information to the analysis, these were considered redundant and not taken into account. Thus, out of the 2,323 single genotypes identified in Laucou *et al*. [8], 2,096 genotypes presenting no missing data and at least three allele differences were analyzed in our study for further structure and clustering analyses (full list and characteristics are given in Additional file 1: Table S1).

### Statistical analysis

Main diversity statistics for the 20 microsatellite markers, such as the total number of alleles, expected heterozygosity and total gene diversity [58] were calculated using Genetix [59]. A private allele index adjusted for sample bias was estimated with ADZE [60], following a generalized rarefaction approach. Genetic differentiation D_est_ was computed using the SMOGD software [61,62], based on the method of Jost [63]. Confidence intervals were calculated using 1000 bootstraps, Polymorphism Information Content (PIC) according to Botstein *et al*. [64], and the single parent exclusion probabilities according to Jamieson & Taylor [65].

The dataset of 2,096 unique genotypes was used to run a series of tests, with the Bayesian method implemented in STRUCTURE [27], in order to find the best model to infer population structure (with or without admixture, correlated allele frequencies, or prior information about sampling locations) and the best K_s_ level of population subdivision, with K_s_ varying from 2 to 12. Within STRUCTURE, we allowed an iterative process with a burn-in phase of 5 × 10^4^ iterations, and a sampling phase of 5 × 10^4^ replicates. Ten replicates of each assumed K_s_-level subdivision were compared to estimate group assignation stability.

We first evaluated the different models of admixture, allele frequencies and prior population information available in STRUCTURE. The most appropriate model to interpret our data appeared to be the uncorrelated allele frequencies and prior geographic information model, which showed a better stability between runs for K_s_ = 3 and 5, and a lower variance for K_s_ = 5, as compared to other possible STRUCTURE models (Additional file 3: Figure S1).

Since the geographic groups are not equally represented in Vassal, a second analysis was run to measure a possible sampling effects, in particular the bias that could arise from oversampling one region or one family group. This was tested by running STRUCTURE on two different set of genotypes (Table 1), the full set of 2096 genotypes (*set1*) and a sub-set of 888 genotypes randomly drawn to constitute equally-sized, geographic origin-based groups (*set2*).

Finally, the most probable uppermost level of structure subdivision between the successive K_s_ values was estimated with two methods: 1) the calculation of Evanno’s delta-K as the second order change in the likelihood function divided by the standard deviation of the likelihood [66,67], and 2) the similarity coefficient between each pair of runs, which provides an evaluation of the stability of the solutions between runs.

Genotypes were assigned to a cluster when 85% or more of their inferred genome belonged to the cluster, the genotypes with a lower score being considered as “admixed”. The chosen clusters for each Ks level were then labeled according to a three digit code (e.g. S-2.1, S-2.2,…, S-5.5) for further geographic and phenotypic characterization. A graphical display of the individual and group distances was obtained with a Principal Component Analysis (PCA) using the package *adegenet* implemented in R [68,69].

To validate the STRUCTURE clustering, we compared its output with that obtained using a less constrained method of clustering. Odong *et al*. [30] highlighted that STRUCTURE and Ward’s method [28] are convergent and complementary. Thus we used Ward’s method to evaluate the distances between clusters minimizing the sum of squares of any two clusters at each step. Using Ward dissimilarity matrix, we built a dendrogram with DARwin software [29]. The advantage of Ward clustering is to provide details of the relationships at any level, as close as family levels. One disadvantage is that it does not deal with admixed genotypes. We indexed the Ward subdivision levels as K_w_, and labeled the subgroups accordingly (W-2.1, W-2.2,…, W-12.12).

### Genetic structure partitioning between and within groups - Family relationships

To estimate the part of the population genetic structure due to parentage, we first calculated the most probable family relationship among each pair of genotypes using the ML-relatedness software [70]. Genotype pairs (half of a 2,096 x 2,096 matrix minus the diagonal [(n^2^-n)/2)] = 2,195,569 couples) were declared either unrelated or family-related, this latter category grouping full sibs, half-sibs, and parent-offsprings, in order to lower the chance of false attribution. Only non-ambiguous relationship assignations (according to 99.9% confidence intervals calculated in 100 mating population simulations) with an experimentally determined LOD score > 9 were taken into account. For each of the above subdivisions we also calculated a weighted average relatedness *r*^*2*^.

The results of this analysis were then assigned to categories of STRUCTURE subdivision (e.g. within or among subgroups), according to the group of each parent. Within-group average relatedness was estimated with the formula of Queller and Goodnight [71] and its standard deviation with 100 jackknifes over loci, using the RERAT software [72].

### Phenotypic evaluation of the different K levels

To interpret the population structure in terms of cultivar utilization, movement and history, each subpopulation was finally characterized for its flower and fruit traits and for its geographic origin. Group names were ultimately based on their main characteristics.

## Competing interests

The authors declare that they have no competing interests.

## Authors’ contributions

RB, JPP, TL, MDVS, and LL carried out the statistical calculations. VL carried out the molecular analysis. LL, MDVS, PT, RB and JPP suggested and discussed statistical methods. TL and JMB took care of, identified and classified the plant material, and interpreted the statistical results with regard to the history of viticulture. BG managed the genetic resources’ collection and provided the plant material. RB wrote the paper with the help and corrections of TL, LL, JMB, JPP, and PT. PT conceived the study and was PhD work coordinator for MDVS. JMB supervised the work. All authors read and approved the final manuscript.

## Authors’ information

Thierry Lacombe, Blaise Genna: (http://www1.montpellier.inra.fr/vassal/)

## Supplementary Material

Additional file 1: Table S1 List of the 2096 genotypes (introduction and variety names, cultivar code) and their main characteristics (geographic origin, utility, sex, skin color, flesh color, berry flavor and seeds). Click here for file

Additional file 2: Table S2 Diversity indexes for the 20 microsatellite loci and the 2,096 cultivated grapevine genotypes. Click here for file

Additional file 3: Figure S1 The four steps of the graphic method of Evanno *et al*. (2005), allowing the estimation of the true number of ancestral groups K. Click here for file

Additional file 4: Table S3 Geographic and agro-technological characterization of the STRUCTURE subgroups. Click here for file

Additional file 5: Figure S2 Full dendrogram of the 2096 cultivated grapevines, according to a clustering based on the 20 SSR marker data and the Ward method. The short name of the varieties was used (use the zoom option to read). Group color codes and numbers are given in the Additional file 1, **Table S1**, "codes" sheet. Click here for file

Additional file 6: Table S4 Comparison of the clustering between the STRUCTURE and the Ward methods. Click here for file

Additional file 7: Table S5 Geographic and agro-technological characterization of the Ward subgroups. Click here for file

Additional file 8: Table S6 Matrix of the D_*est*_ among STRUCTURE groups. Click here for file

Additional file 9: Table S7 Group agro-morphological characterization. Click here for file
