# Supplementary material for: Genetic structure in cultivated grapevines is linked to geography and human selection
Source: BMC Plant Biol. 2013 Feb 8;13:25. doi: 10.1186/1471-2229-13-25 (PMC3598926; doi:10.1186/1471-2229-13-25)
Supplement: Additional file 2: Table S2 — Diversity indexes for the 20 microsatellite loci and the 2,096 cultivated grapevine genotypes. [file 1471-2229-13-25-S2.doc]

Supplementary Table S2. Diversity indexes for the 20 loci and the 2096 cultivated grapevine genotypes.

| **Locus name** | **Number of Alleles** | **Most frequent allele** | **Unbiased exp H. (*)** | **Observed heterozygosity** | | **PIC (**)** | | **SP. exclusion p. (***)** | |
| --- | --- | --- | --- | --- | --- | --- | --- | --- | --- |
| VMC1b11 | 14 | 30% | 0.825 | 0.835 | | 0.8049 | | 0.4985 | |
| VMC4f3 | 29 | 28% | 0.868 | 0.838 | | 0.8569 | | 0.6075 | |
| VVIb01 | 11 | 40% | 0.666 | 0.658 | | 0.6072 | | 0.2501 | |
| VVIh54 | 21 | 42% | 0.744 | 0.736 | | 0.7131 | | 0.3782 | |
| VVIn16 | 5 | 44% | 0.688 | 0.654 | | 0.6405 | | 0.2664 | |
| VVIn73 | 7 | 79% | 0.361 | 0.344 | | 0.3471 | | 0.0710 | |
| VVIp31 | 18 | 17% | 0.892 | 0.895 | | 0.8818 | | 0.6443 | |
| VVIp60 | 18 | 37% | 0.749 | 0.763 | | 0.7149 | | 0.3774 | |
| VVIq52 | 8 | 44% | 0.672 | 0.658 | | 0.6107 | | 0.2598 | |
| VVIv37 | 18 | 34% | 0.827 | 0.767 | | 0.8101 | | 0.5148 | |
| VVIv67 | 34 | 23% | 0.851 | 0.780 | | 0.8351 | | 0.5655 | |
| VVMD21 | 9 | 49% | 0.686 | 0.681 | | 0.6494 | | 0.2960 | |
| VVMD24 | 10 | 50% | 0.674 | 0.700 | | 0.6348 | | 0.2927 | |
| VVMD25 | 16 | 28% | 0.784 | 0.806 | | 0.7495 | | 0.4132 | |
| VVMD27 | 12 | 23% | 0.826 | 0.843 | | 0.8032 | | 0.4946 | |
| VVMD28 | 24 | 18% | 0.883 | 0.870 | | 0.8722 | | 0.6159 | |
| VVMD32 | 21 | 31% | 0.827 | 0.822 | | 0.8071 | | 0.5026 | |
| VVMD5 | 14 | 22% | 0.861 | 0.856 | | 0.8457 | | 0.5600 | |
| VVMD7 | 18 | 30% | 0.810 | 0.808 | | 0.7868 | | 0.4720 | |
| VVS2 | 17 | 28% | 0.838 | 0.819 | | 0.8207 | | 0.5374 | |
| **Average (sd)** | **16.1**  **(7.34)** | **--** | **0.7666 (0.1219)** | **0.7567**  **(0.1226)** | | ***Cumulative*** | | ***0.999993*** | |
| (*) Unbiased expected heterozygosity (Nei 1987) | | | | |  | |  | |  |
| (**) Polymorphism information content (Botstein et al. 1980) | | | | |  | |  | |  |
| (***) Single parent exclusion probabilities (Jamieson & Taylor 1997) | | | | | | |  | |  |
