# Supplementary material for: Genetic structure in cultivated grapevines is linked to geography and human selection
Source: BMC Plant Biol. 2013 Feb 8;13:25. doi: 10.1186/1471-2229-13-25 (PMC3598926; doi:10.1186/1471-2229-13-25)
Supplement: Additional file 3: Figure S1 — The four steps of the graphic method of Evanno et al. (2005), allowing the estimation of the true number of ancestral groups K. [file 1471-2229-13-25-S3.doc]

***Supplementary Figure S1***


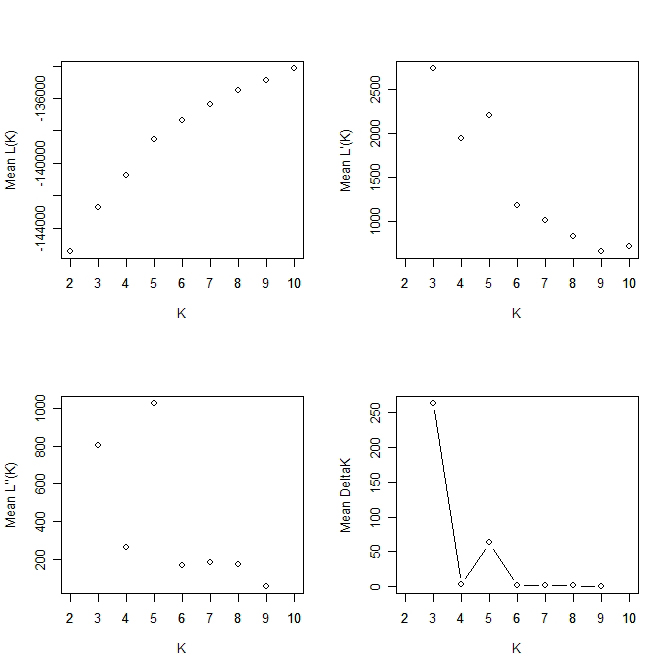


*Figure S1: The four steps of the graphical method of Evanno et al. (2005), allowing the estimation of the true number of ancestral K groups.*
