# Supplementary figures and images for: Genetic structure in cultivated grapevines is linked to geography and human selection
Source: BMC Plant Biol. 2013 Feb 8;13:25. doi: 10.1186/1471-2229-13-25 (PMC3598926; doi:10.1186/1471-2229-13-25)

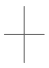

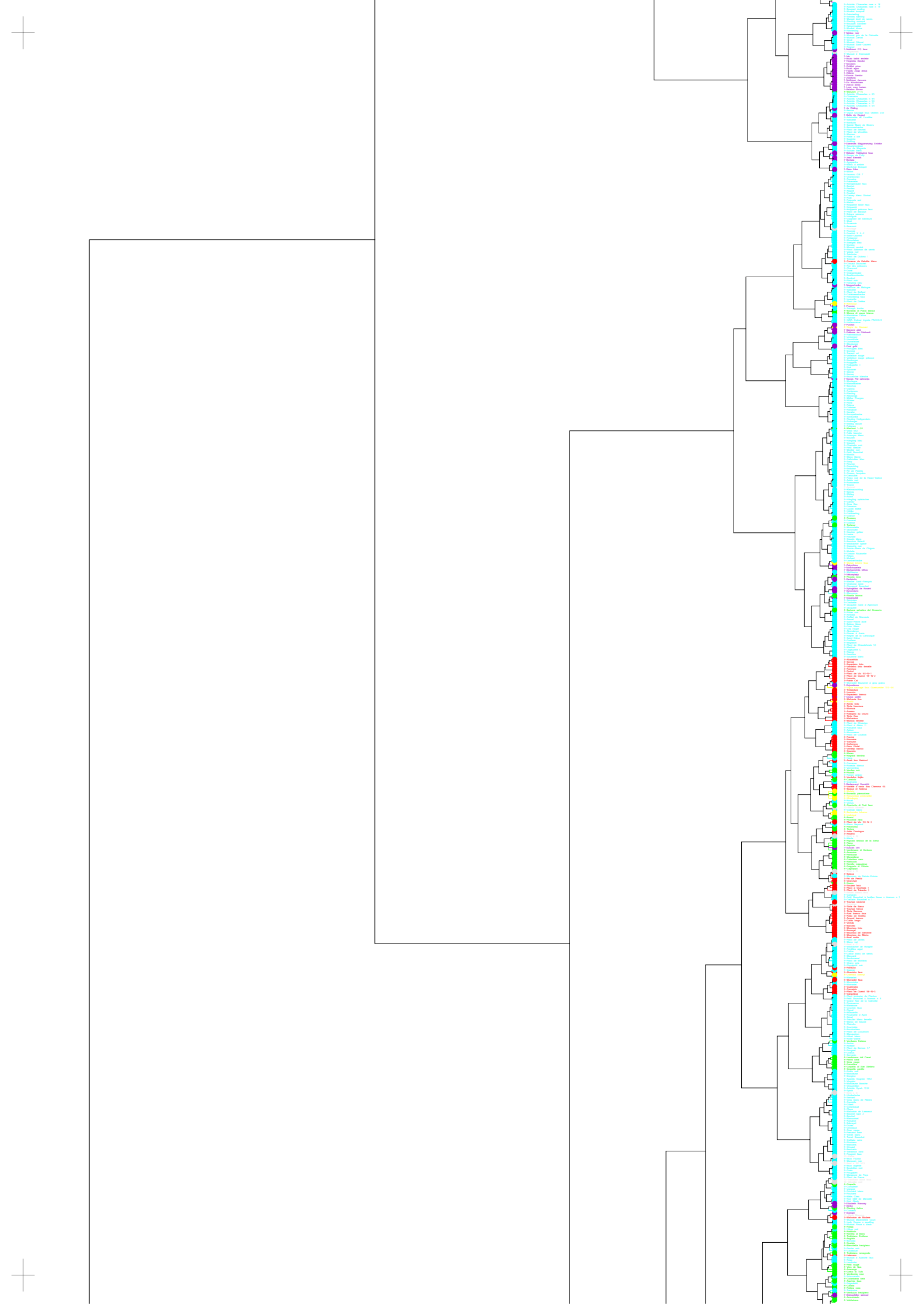

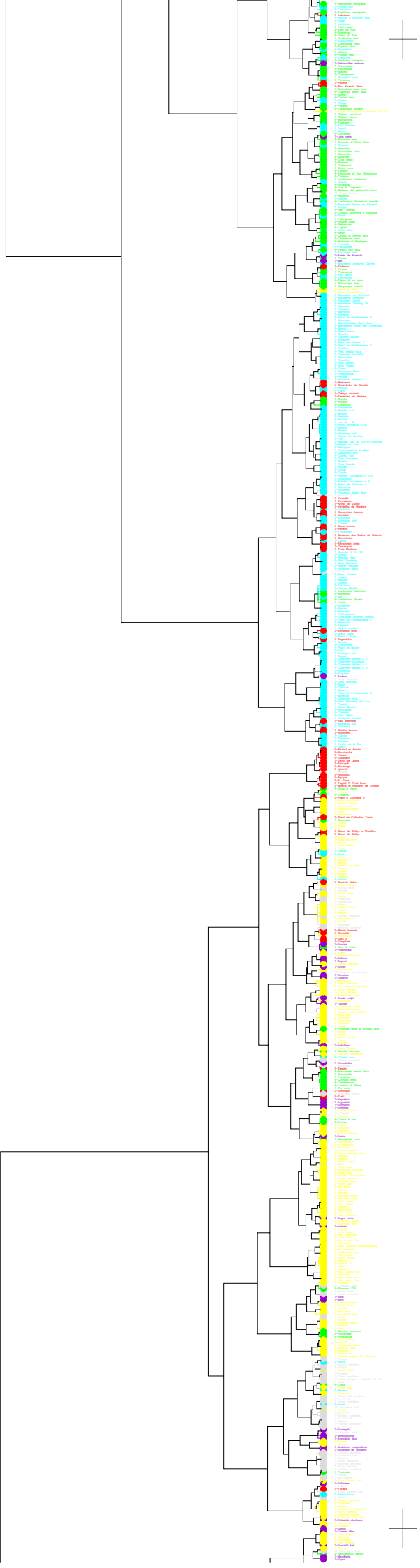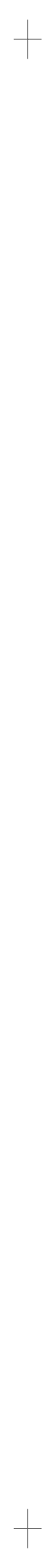

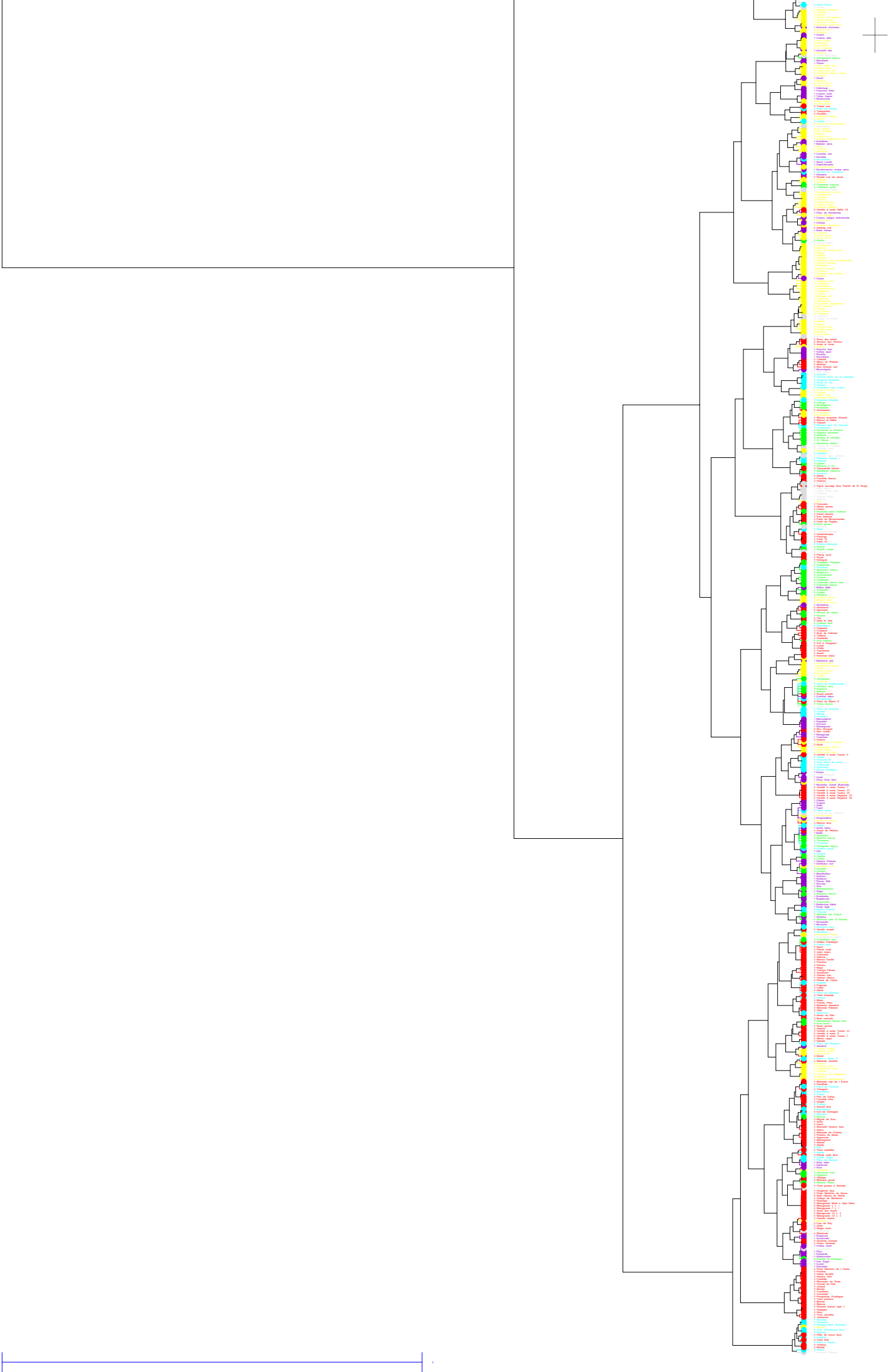

Supplement: Additional file 5: Figure S2 — Full dendrogram of the 2096 cultivated grapevines, according to a clustering based on the 20 SSR marker data and the Ward method. The short name of the varieties was used (use the zoom option to read). Group color codes and numbers are given in the Additional file 1, Table S1, "codes" sheet. [file 1471-2229-13-25-S5.pdf]
