# Supplementary material for: Genetic structure in cultivated grapevines is linked to geography and human selection
Source: BMC Plant Biol. 2013 Feb 8;13:25. doi: 10.1186/1471-2229-13-25 (PMC3598926; doi:10.1186/1471-2229-13-25)
Supplement: Additional file 6: Table S4 — Comparison of the clustering between the STRUCTURE and the Ward methods. [file 1471-2229-13-25-S6.doc]

*Supplementary Table S4*. Comparison of the clustering among the STRUCTURE and the Ward methods.

|  | Ward groups (K_w_3) | **W-3.1** | **W-3.2** | **W-3.3** |  | |
| --- | --- | --- | --- | --- | --- | --- |
| *STRUCTURE* Groups (K_s_3) | N. of genotypes | 719 | 797 | 580 |  | |
| **S-3.1** | 419 | **372** | 7 | 40 |  | |
| **S-3.2** | 356 | 4 | **342** | 10 |  | |
| **S-3.3** | 226 | 14 | 23 | **189** |  | |
| *Non-Attributed* | *1095* | *329* | *425* | *341* |  | |
|  |  |  |  |  |  | |
|  |  |  |  |  |  |  |
|  | Ward groups (K_w_5) | **W-5.1** | **W-5.2** | **W-5.3** | **W-5.4** | **W-5.5** |
| *STRUCTURE* Groups (K_s_5) | N. of genotypes | 521 | 276 | 719 | 360 | 220 |
| S-5.1 | 97 | **82** | 4 | 5 | 5 | 1 |
| **S-5.2** | 153 | 33 | **120** |  |  |  |
| **S-5.3** | 298 | 4 |  | **281** | 12 | 1 |
| S-5.4 | 104 |  |  | 1 | **101** | 2 |
| **S-5.5** | 165 | 19 | 1 | 20 |  | **125** |
| *Non-Attributed* | 1279 | 383 | 151 | 412 | 242 | 91 |
|  |  |  |  |  |  |  |
