# Supplementary material for: Genetic structure in cultivated grapevines is linked to geography and human selection
Source: BMC Plant Biol. 2013 Feb 8;13:25. doi: 10.1186/1471-2229-13-25 (PMC3598926; doi:10.1186/1471-2229-13-25)
Supplement: Additional file 9: Table S7 — Group agro-morphological characterization. [file 1471-2229-13-25-S9.doc]

# *Supplementary Table S7*

# *Agro-morphological traits of the clusters*

# A) *STRUCTURE* groups

# Version du 4 octobre 2010

*Subdivision level K_s_= 3*

Subgroup S-3.1 : Black and white wine cultivars from western and central Europe (Portugal to Germany and mostly France), with the presence of important kin groups of Pinot, Gouais and Savagnin.

Subgroup S-3.2 : Traditional table grape cultivars originated from far and middle East to central Asia and new selections of table grape cultivars, with mainly white, red, grey or pink berry skin colour ; this group gathers most of the seedless grapevine and several female cultivars.

Subgroup S-3.3 : Wine cultivars from Eastern Europe and Balkan peninsula, with mostly white berry skin colour ; in this group several Muscat flavour cultivars and female cultivars may also be found.

*Subdivision level K_s_= 5*

Subgroup S-5.1 : Wine and table grape cultivars from Iberian peninsula and Maghreb, with mostly white berry skin colour.

Subgroup S-5.2 : Traditional table grape cultivars originated from far and middle East and Caucasus, with mainly white, red, grey or pink berry skin colour and neutral flavour ; this group gathers most of the seedless grapevine and several female cultivars.

Subgroup S-5.3 : Black and white wine cultivars from western and central Europe (South West France to Alps and Germany) with the presence of important kin groups of Pinot, Gouais and Savagnin.

Subgroup S-5.4 : Table grape cultivars, new breeding or modern selections, with mostly white, red, grey or pink berry skin colour ; this group gathers also most of the Muscat flavour cultivars (presence of Muscat d’Alexandrie progeny).

Subgroup S-5.5 : Wine cultivars from Balkan peninsula and central Europe, with mostly white berry skin colour ; in this group several female cultivars may also be found.

**B) Ward groups**

*Subdivision level K_w_= 12*

Subgroup W-12.1 : Black and white wine cultivars from Western Europe, with a presence of kin group of Gouais blanc.

Subgroup W-12.2 : Black and white wine cultivars from South West of France (and Portugal), with a presence of kin groups of Savagnin and Cabernet franc.

Subgroup W-12.3 : Wine cultivars from Central Europe and Balkan peninsula, with mostly white, red, grey or pink berry skin colour ; in this group several female cultivars may also be found.

Subgroup W-12.4 : Black and white wine cultivars from Iberian Peninsula and Maghreb.

Subgroup W-12.5 : Black and white cultivars (mostly for wine but also for table grape) from Italy and Alps.

Subgroup W-12.6 : Table grape cultivars, new breeding or modern selections, with mostly white berry skin colour and several cultivars with muscat flavour.

Subgroup W-12.7 : Traditional breeding of table grape (including some cultivars with Muscat flavour) selected in several countries during XIXth century and early XXth.

Subgroup W-12.8 : Wine cultivars with white, red, grey or pink berry skin colour. Presence of several cultivars with muscat flavour and kin groups of Chasselas and Muscat à petits grains.

Subgroup W-12.9 : A majority of white wine cultivars from Western Europe with a presence of kin group of Pinot and Riesling and recent crossbreds from Germany.

Subgroup W-12.10 : Wine cultivars with mostly black berry skin colour, from Italy (centre and north), France (Alps and south), Spain (north) and Portugal.

Subgroup W-12.11 : Traditional table grape cultivars originated from far, middle and near East, with mainly white, red, grey or pink berry skin colour ; this group gathers most of the seedless grapevine (kin group of Sultanine including new selections) and several female cultivars.

Subgroup W-12.12 : Diverse wine and table grape cultivars in particular from Caucasus, Russia and Maghreb ; in this group several female cultivars may also be found.
